# Zeaxanthin: Review of Toxicological Data and Acceptable Daily Intake

**DOI:** 10.1155/2016/3690140

**Published:** 2016-01-13

**Authors:** James A. Edwards

**Affiliations:** NIC-RD/HN Toxicology and Kinetics, DSM Nutritional Products Ltd., Wurmisweg 576, 4303 Kaiseraugst, Switzerland

## Abstract

Zeaxanthin is a nutritional carotenoid with a considerable amount of safety data based on regulatory studies, which form the basis of its safety evaluation. Subchronic OECD guideline studies with mice and rats receiving beadlet formulations of high purity synthetic zeaxanthin in the diet at dosages up to 1000 mg/kg body weight (bw)/day, and in dogs at over 400 mg/kg bw/day, produced no adverse effects or histopathological changes. In developmental toxicity studies, there was no evidence of fetal toxicity or teratogenicity in rats or rabbits at dosages up to 1000 or 400 mg/kg bw/day, respectively. Formulated zeaxanthin was not mutagenic or clastogenic in a series of* in vitro* and* in vivo* tests for genotoxicity. A 52-week chronic oral study in Cynomolgus monkeys at doses of 0.2 and 20 mg/kg bw/day, mainly designed to assess accumulation and effects in primate eyes, showed no adverse effects. In a rat two-generation study, the NOAEL was 150 mg/kg bw/day. In 2012, this dosage was used by EFSA (NDA Panel), in association with a 200-fold safety factor, to propose an Acceptable Daily Intake equivalent to 53 mg/day for a 70 kg adult. The requested use level of 2 mg/day was ratified by the EU Commission.

## 1. Introduction

Zeaxanthin (3, 3′-dihydroxy-*β*-carotene, CAS number 144-68-3) is a nutritional carotenoid in a category referred to as xanthophylls. Zeaxanthin is structurally closely similar to lutein. The intake of both carotenoids in the human diet is regarded as healthy, with these components reflecting an adequate intake of fruit and vegetables.

**Figure 1:**
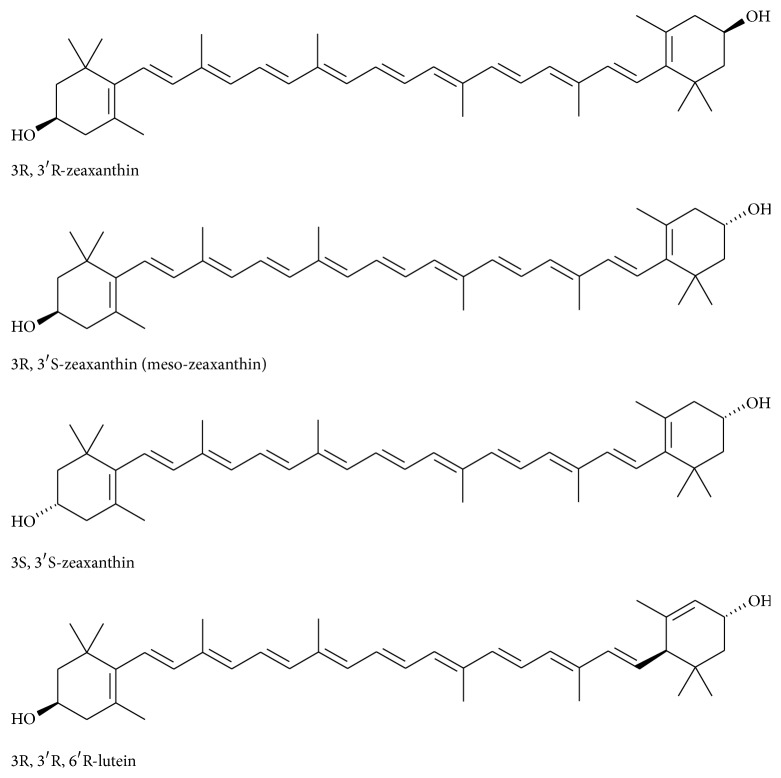
Structures for optical isomers of all-trans zeaxanthin and lutein.

Lutein as a human dietary supplement is often obtained as an extract from* Tagetes* (marigold) and the extract always contains some zeaxanthin. Zeaxanthin itself, on the other hand, tends to be produced from both biological sources and in a highly pure form synthetically. The predominant zeaxanthin stereoisomer in nature and consequently in the diet is the 3R, 3R′-stereoisomer, which is also the predominant stereoisomer of synthetic zeaxanthin (Figure 1).

In normal human food sources, lutein is more abundantly present than zeaxanthin, for example, in spinach, but there are other food sources with a relatively higher content of zeaxanthin, such as egg yolk, corn (maize), or orange pepper [1, 2]. The usual dietary ratio of lutein :  zeaxanthin is approximately 5 : 1 (Table 1) [3, 4].

A closely related stereoisomer that is rarer than 3R, 3′R zeaxanthin isomer in nature is the 3R, 3′S stereoisomer, commonly referred to as meso-zeaxanthin. This stereoisomer, like lutein and zeaxanthin, is found in the human macula and its source has been determined in primates fed with a zeaxanthin-free diet to be derived from lutein [5, 6].

In addition to being generally healthy and acting as antioxidants, a specific protective activity exists in the eyes of primates. In the primate eye, in the center of the retina, an area known as the macula lutea is visible as a yellow spot due to the accumulation of the macular xanthophylls. The presence of the xanthophyll carotenoids in the human appears to be physiologically significant; the concentration of xanthophylls in the macula is the highest concentration found everywhere in the primate body. Furthermore, based on filtration of potentially damaging light and quenching of photochemically induced reactive oxygen species, it is believed [7, 8] that, via these mechanisms, lutein, zeaxanthin, and meso-zeaxanthin may contribute to reducing the risk of Age-Related Macular Degeneration (AMD), a leading cause of irreversible loss of vision observed in western countries.

There is a considerable amount of safety data for zeaxanthin based mainly on routine regulatory studies with high purity synthetic zeaxanthin, manufactured by DSM Ltd. (previously manufactured by F. Hoffmann-La Roche Ltd.). A series of* in vitro* and* in vivo* tests for genotoxicity have been undertaken as well as subchronic safety studies (13 weeks in duration) by dietary exposure at high dosage levels in mice, rats and dogs. Developmental toxicity studies have been undertaken in rats and rabbits, a two-generation study was performed on the rat, and a chronic study of 52 weeks' duration was performed on Cynomolgus monkeys. ADME (absorption, distribution, metabolism, and excretion) studies have been undertaken. These studies with zeaxanthin are reviewed here.

Potentially, data for certain closely related substances may have relevance or should be taken into consideration, in the safety evaluation of zeaxanthin. The inclusion of a ferret study with the related xanthophyll, *β*-cryptoxanthin, on a read-across basis, to address the question if zeaxanthin consumption might have an adverse impact on cigarette smokers, is described. Reference is also made to known studies with lutein and meso-zeaxanthin.

Safety data from human intervention studies in which synthetic zeaxanthin has been supplemented, of which the AREDSII study is by far the largest, is also considered and the apparent safe level of intake from these studies compared with that derived from the animal studies.

The safety data for lutein have been evaluated by the European Food Safety Authority (EFSA). Due to the close similarity of lutein and zeaxanthin, it is probable that the toxicology for the pure substances is very similar, although it has to be remembered that within the eye a highly specific biological stereoisomeric differentiation may occur. Many of the analytical methods used in the past did not differentiate zeaxanthin and lutein such that the information on the differential occurrence of lutein and zeaxanthin in fruits and vegetables for many years was incomplete. The Joint FAO (Food and Agriculture Organization of the United Nations)/WHO (World Health Organization) Expert Committee on Food Additives (JECFA) in 2006 [9] in their safety evaluation of lutein and zeaxanthin defined a “group” ADI (Acceptable Daily Intake) for lutein and zeaxanthin of 0–2 mg/kg bw/day, covering both substances.

The toxicity of compounds can often be strongly influenced by their purity. If coming from a natural source, the other natural components or contaminants (e.g., pesticides) need to be taken into consideration. If coming from chemical synthesis, a representative batch of typical purity containing the synthetic by-products that might be present needs to be tested. In the regulatory studies for zeaxanthin presented here coming from DSM Nutritional Products Ltd., the zeaxanthin tested was in accord with the manufacturer's purity specification of at least 98% zeaxanthin (>96% all-trans, <2% cis). This high purity substance is marketed in a formulation designed to provide stability against oxidation and enhance bioavailability.

The DSM studies reported here have been undertaken by a number of toxicologists and safety specialists over a number of years and are described mainly in summary form. The individual study reports describe the results in detail. In the regulatory processes to obtain approval for human use, the detailed reports are supplied to the respective regulatory authority.

## 2. Methods: Regulatory Animal Safety Studies with Synthetic Zeaxanthin

An extensive array of conventional toxicology studies has been undertaken with DSM synthetic zeaxanthin (Ro 01-9509). The studies undertaken by DSM were for the purpose of assessing safety from oral intake, or for worker safety purposes, and are listed in Table 2.

The toxicology studies undertaken by DSM were predominantly undertaken using recognized international regulatory guidelines and, in particular, respective Organization for Economic Cooperation and Development (OECD) guidelines. The OECD guideline stipulates for each study design how the study should be performed with a detailed range of study design requirements, such as numbers of replicates or animals, the concentrations or dosages that are appropriate to ensure sufficiently stringent testing, details of the endpoints that should be investigated, and guidance on the evaluation of the data obtained.

In the case of the two-generation rat study, the key regulatory study for the overall safety assessment, the study was designed to meet the known requirements of the OECD 416 guidelines (22 January, 2001) and the US FDA Center for Food Safety and Applied Nutrition, Redbook 2000, Toxicological Principles for the Safety of Food Ingredients, IV.C.9.a. Guidelines for Reproduction Studies (20 July, 2000).

Additionally, the studies were undertaken following the principles of Good Laboratory Practice (GLP). In the case of the two-generation rat study, the study was conducted in accordance with the OCD GLP guideline and UK GLP guidelines, as the study was conducted in UK. Analyses undertaken to confirm the stability and concentrations of zeaxanthin in the treated diet as well as analyses undertaken to assess the concentration of zeaxanthin in plasma and liver samples were performed at the DSM test site in Switzerland and performed in accordance with Swiss ordinance on GLP.

Additionally,* in vivo* studies were undertaken following the local national requirements on animal housing and animal welfare requirements such as in the UK, the Home Office “code of practice for the housing and care of animals used in scientific procedures.”

Importantly, the evaluation of zeaxanthin involved a special study on Cynomolgus monkeys, which included a range of specific endpoints to investigate safety to the primate and human eye. The Cynomolgus monkey has been shown to be an excellent model to investigate the induction and dose dependency of canthaxanthin crystal formation in the retina [10–13]. Using similar procedures as described in these publications, the study included indirect ophthalmoscopic examinations performed using the Bonnoskop and direct ophthalmoscope and a contact lens biomicroscope. Additional evaluations were performed using the ophthalmic slit lamp biomicroscope in combination with wide-field corneal contact fundus lenses. Electroretinography (ERG) was undertaken at intervals during the study. Terminal eye pathology involved evaluation of whole-mounts of retinas from the right eyes by microscopic investigation with light or confocal microscopy. Maculas were investigated under the polarization microscope and semiquantitative analysis of inclusions was performed. Routine histopathological investigation of paraffin sections from retinal periphery was performed and zeaxanthin and lutein in the retina and lens were measured analytically by High-Performance Liquid Chromatography (HPLC).

## 3. Results

### 3.1. Genotoxicity Studies

#### 3.1.1. *S. typhimurium* Mutagenicity (Ames) Test, OECD 471

Crystalline zeaxanthin was evaluated for mutagenic activity in the Ames assay using the plate incorporation and the preincubation method. Seven* Salmonella typhimurium* standard tester strains were employed (TA1535, TA1537, TA1538, TA97, TA98, TA100, and TA102) with and without an exogenous metabolic activating enzyme system (S9-mix) derived from livers of phenobarbital/*β*-naphthoflavone treated male rats. Due to the strong precipitation of the test compound in the aqueous medium, 1500 *μ*g/plate was chosen as the highest dose level. There was no increase of the numbers of mutants in any of the tester strains, while the positive controls verified the sensitivity of the strains and the activity of the S9-mix [14].

In one very early laboratory batch of pure crystalline zeaxanthin, a positive result was found in the Ames test. It was determined that pure zeaxanthin is not mutagenic; however, degradation products formed during exposure of crystalline zeaxanthin to air and light were considered responsible for the mutagenic activity [15]. In addition, it was determined that components in the beadlet formulation scavenged the mutagenic activity of degraded crystalline zeaxanthin, thus further protecting against the occurrence of mutagenic activity. The beadlet formulation for the marketed product includes the antioxidants (ascorbyl palmitate, sodium ascorbate, and* dl*-*α*-tocopherol), which prevent degradation.

Crystalline zeaxanthin that had been kept in storage beyond the maximum shelf life was subsequently assessed in a non-GLP Ames test [16]. The purpose of this study was to confirm the absence of mutagenic activity of crystalline zeaxanthin. Five* Salmonella typhimurium* tester strains (TA1535, TA97, TA98, TA100, and TA102) were employed with and without metabolic activation (S9-mix). No relevant increase in the number of revertant colonies was apparent and it was concluded that neither zeaxanthin nor any of the metabolites formed by the metabolic activation system was mutagenic in the Ames test.

#### 3.1.2. Gene Mutation Assay in V79/HGPRT Cells, OECD 476

In the gene mutation assay in cultured mammalian cells, zeaxanthin was tested for its ability to induce gene mutations at the HGPRT (Hypoxanthine Guanine Phosphoribosyl Transferase) locus in the established cell line V79, derived from Chinese hamster lung cells. Treatment with 1 *μ*g to 16 *μ*g/mL (0.002–0.03 mmol/L) did not induce mutations to 6-thioguanine resistance in V79 cells* in vitro*, neither in the absence nor in the presence of a rat liver activation system [17].

#### 3.1.3. Unscheduled DNA Synthesis (UDS) Assay, OECD 482

The ability of zeaxanthin to induce DNA damage was tested by the Unscheduled DNA Synthesis assay (UDS test) as measured by the incorporation of radiolabeled nucleotides into nonreplicated DNA of freshly isolated rat hepatocytes. A 20-hour exposure to 1 *μ*g to 16 *μ*g zeaxanthin per mL did not induce DNA repair synthesis in primary cultures of rat hepatocytes [18].

#### 3.1.4. Chromosome Analysis of Human Peripheral Lymphocytes, OECD 473

The potential* in vitro* clastogenic activity of zeaxanthin was assessed using human peripheral blood lymphocytes as target cells in the presence and absence of rat liver activating enzyme system (S9-mix). Under the experimental conditions described, neither zeaxanthin nor any of its metabolites induced chromosomal aberrations in human peripheral blood lymphocytes [19].

#### 3.1.5. Mouse Micronucleus Assay, OECD 474

Zeaxanthin was tested in the* in vivo* micronucleus assay in mice. Zeaxanthin, 10% beadlet formulation, was administered orally at dose levels of 44.5, 89.0, and 178 mg/kg of zeaxanthin 30 and 6 hours prior to sacrifice. There was no increase of micronuclei; thus, it was concluded that under the conditions of the study zeaxanthin did not induce chromosome breaks or mitotic nondisjunctions in mouse bone marrow cells at doses up to 178 mg/kg of zeaxanthin [20].

### 3.2. Short-Term Toxicity Studies

#### 3.2.1. Acute Safety Studies, Pre-OECD Guideline, Similarities to Guideline OECD 401

Acute studies with zeaxanthin were performed in rats and mice. Zeaxanthin has a low order of acute toxicity. All mice and rats survived a single oral dose of up to 4000 mg/kg in rats and 8000 mg/kg in mice. The LD_50_ values in rats and mice, therefore, were greater than 4000 and 8000 mg/kg body weight, respectively [21].

#### 3.2.2. Guinea Pig Skin Sensitization Test, OECD 406

An optimization test (according to Maurer) was performed with zeaxanthin in albino guinea pigs of both sexes. No signs of skin irritation or sensitization were observed [22]. A subsequent maximization test in albino guinea pigs, based on OECD guideline 406, was also negative [23].

#### 3.2.3. Rabbit Irritation Test, OECD 405

The primary eye irritation potential of zeaxanthin was studied in young adult rabbits [24]. The risk that an accidental or occasional ocular exposure to zeaxanthin could cause injury to the eye in man was considered to be low.

### 3.3. General Toxicology Studies

#### 3.3.1. Subchronic Safety Studies

13-week subchronic toxicity studies have been performed with synthetic zeaxanthin in three species, mouse, rat, and dog. Preliminary studies (5- and 10-day studies) were conducted beforehand to ensure appropriate selection of dosages for the main studies.

#### 3.3.2. 13-Week Study in Mice, Similar to OECD 408

A 13-week oral safety study was performed in mice with a 9.3% beadlet formulation of zeaxanthin, administered as a feed admixture. Groups of 10 male and 10 female mice were treated with 0, 250, 500, and 1000 mg/kg body weight/day (mg/kg bw/day) of zeaxanthin. The placebo beadlets were added to the diet so that all 4 groups received similar amounts of beadlets. There was no treatment-related hematology or clinical chemistry findings. No discoloration of adipose tissue or other findings were observed at necropsy and there were no histopathological effects attributable to zeaxanthin or the beadlet formulations. The No Observed Adverse Effect Level (NOAEL) of zeaxanthin was >1000 mg/kg bw/day in mice [25].

In line with the respective OECD guideline procedures for mice, the study did not include ophthalmoscopy, although histopathology of the eyes was undertaken.

#### 3.3.3. 13-Week Study in Rats, OECD 408

An original 13-week oral safety study was conducted in rats with a 9.3% beadlet formulation of zeaxanthin administered as a feed admixture. Groups of 16 male and 16 female rats were treated with 0, 250, 500, and 1000 mg/bw/day of zeaxanthin. The NOAEL for zeaxanthin was >1000 mg/kg bw/day in rats [26].

Due to a change in manufacturing process, a second 13-week oral safety study in rats was performed with a 10% beadlet formulation of zeaxanthin from an updated process. Groups of 16 male and 16 female rats were treated with doses of 0, 250, 500, and 1000 mg/kg bw/day of zeaxanthin as a dietary admixture [27]. All groups received similar amounts of beadlets, by adjusting the diet with control beadlets. There was no effect of treatment on food intake and body weight. Yellow-orange discoloration of the feces was seen in all zeaxanthin-treated rats, especially at the high dose. No treatment-related changes in hematological and clinical chemistry parameters were observed. Urine pH values were slightly decreased in male rats of all dose groups. In line with the respective OECD guideline, the study included ophthalmoscopic evaluations. About 20–30 minutes prior to examination, a mydriatic was instilled into each eye of control and high-dose animals. The examinations were made using a “KEELER” Fison binocular ophthalmoscope. There were no treatment-related changes.

At necropsy, a slight orange discoloration of the adipose tissue was reported in all treated animals; however, this was not considered an adverse effect but due to color of the test compound. There were no treatment-related changes in organ weights or histopathological findings. Under the conditions of this study, the NOAEL in this second rat study was again >1000 mg/kg bw/day.

#### 3.3.4. 13-Week Dog Study, Similar to OECD 409

A 13-week safety study in dogs was conducted with a 9.4% beadlet formulation of zeaxanthin. Zeaxanthin beadlets were incorporated into feed pellets and fed to groups of 3 male and 3 female beagle dogs to achieve a dose of zeaxanthin of 0, 123, 204, and 422 mg/kg bw/day (males) and 0, 104, 238, and 442 mg/kg bw/day (females). This corresponds to test article concentrations in feed of 0, 4, 8, and 16%, respectively. Control beadlets were added so that the amount of beadlets present in the feed cubes was similar for all groups.

No treatment-related toxicity was observed throughout the study. The test article was found to strongly discolor and to slightly soften the feces, particularly in the high-dose group. Ophthalmoscopic evaluations were undertaken at day and at the end of week 13. Following induction of mydriasis, eyes including cornea, chambers, lens, and retina of all dogs were examined using a fundus-camera KOWA RC-2. The central parts of the retina (generally including the optic disc) were recorded on an Ektachrome-X film. No treatment-related findings were reported. Urinalysis as well as hematological and serum clinical chemistry investigations showed no treatment-related effects. At necropsy, male dogs from the mid- and high-dose groups showed slight to moderate discoloration (yellow to reddish) in the adipose tissue, which was considered not an adverse effect and probably reflected presence of zeaxanthin. There were no treatment-related histopathological findings. The NOAEL in this study was >422 mg/kg bw/day [28].

### 3.4. Reproductive Safety Studies

#### 3.4.1. Developmental Toxicity Study in Rats, OECD 414

In a developmental toxicity study in rats, zeaxanthin (10% beadlet formulation) was administered at doses of 0, 250, 500, and 1000 mg/kg bw/day orally as a feed admixture from day 7 through day 16 of gestation [29]. A subgroup was Caesarian-sectioned on day 21 of gestation and a rearing subgroup was allowed to deliver naturally and was observed up to day 23 of lactation. There was no indication of any embryotoxic or teratogenic action of zeaxanthin in any of the treated groups. The rearing subgroup showed no indication of any functional abnormalities in the treated groups. It was concluded that, under the conditions of this study, zeaxanthin was neither embryotoxic nor teratogenic in rats at doses up to 1000 mg/kg bw/day.

#### 3.4.2. Developmental Toxicity Study in Rabbits, OECD 414

In a developmental toxicity study in rabbits, zeaxanthin was administered at doses of 0, 100, 200, and 400 mg/kg bw/day orally in rapeseed oil from day 7 through day 19 of gestation. Rabbits were Caesarian-sectioned on day 30 of gestation [30]. No deaths or signs of maternal toxicity were observed in the treated groups. There was no indication of any embryotoxic or teratogenic action of zeaxanthin in the treated groups. There were some isolated malformations among the groups, including controls, but there was no evidence of any treatment-related effect. It was concluded that, under the conditions of this study, zeaxanthin was neither embryotoxic nor teratogenic in rabbits at doses up to 400 mg/kg bw/day.

#### 3.4.3. Two-Generation Study in Rats, OECD 416

A two-generation study was performed with synthetic zeaxanthin in rats [31]. Multigeneration studies involve exposure to test compounds beginning before mating, continuing during mating, and throughout gestation and lactation, until weaning, and cover all reproductive life phases over two generations. There was a range of developmental and behavioral testing in addition to reproduction endpoints. Ophthalmologic examination is not part of the OECD 416 guideline study requirements but testing on the young rats included confirmation of the pupillary reflex response and corneal tactile response.

In the two-generation study, DSM-manufactured zeaxanthin was administered in the diet at nominal doses of 0 (control diet), 0 (placebo beadlet control), 50, 150, and 500 mg/kg bw/day active ingredient, by admixture of 10% WS beadlets to the feed. Although zeaxanthin in a 13-week toxicity study in the rat was well tolerated at 1000 mg/kg bw/day, to achieve a dose level of 1000 mg/kg/day, the beadlet concentration in the diet approached 20%. The high-dose level 500 mg/kg bw/day was selected on the basis of avoiding potential nutritional imbalance due to the high beadlet content in the diet over the duration of a two-generation study. Two control groups received either the control diet only or placebo beadlets incorporated in the diet at the same concentrations as the zeaxanthin beadlets in the high-dose group. The parental (P) generation females were allowed to litter and rear their offspring to weaning. Young were randomly selected from each group to form the filial (F_1_) generation.

Administration of 500 mg/kg bw/day zeaxanthin active ingredient to rats for two successive generations produced marginal adult toxicity in terms of slightly lower food intake during the lactation period of the P generation, a slightly lower body weight gain during the gestation period of the F_1_ generation, and a possible, slight, adverse effect on fertility of the F_1_ generation with a lower mating index (mating index is the number of females with determined copulations/number of oestrous cycles required for their insemination × 100) and slightly fewer pups were born (Table 3). At this dosage in both generations, percentage of pup growth during lactation was also slightly lower than in the control groups (Table 3).

Samples of plasma and liver from the P and F_1_ generation adults and pups were analysed for zeaxanthin exposure monitoring. The analysis results showed that exposure was essentially similar in the P and F_1_ generations. Higher plasma concentrations and notably higher liver concentrations were observed in the weanling pups at day 21 postpartum compared to the adults of the corresponding treatment group (Table 4). Exposure increased with increasing dose although it was not proportional to dose. The higher exposure in the 21-day-old pups probably reflects intake through the maternal milk and that they had already started eating the treated diet provided to the mothers. The relative concentrations in the liver in comparison to concentrations in the plasma were notably higher, in both adults and weanlings (Table 4), suggesting these are accumulation in the liver. However, the high accumulation in the liver of weanlings (3 weeks of age) clearly diminished during subsequent rearing and as adults of the F_1_ generation showed similar tissue concentrations to the P generation, which was exposed from 6 weeks of age.

In conclusion, the NOAEL was defined as the nominal dosage of 150 mg zeaxanthin/kg bw/day.

### 3.5. Chronic Rodent Carcinogenicity Studies

No carcinogenicity studies are available for zeaxanthin, or for lutein. Genotoxicity studies were negative and histological examinations of tissues from repeat dose toxicity studies have not shown any preneoplastic effects or indeed no indication of histological effects at all.

There is data for two carotenoids (canthaxanthin and astaxanthin) showing that chronic administration over two years in rats, but not mice, induces liver toxicity. These two carotenoids induce liver enzyme including cytochrome P450 enzyme CYP1A in the rat [32, 33], although not in the mouse [34] and not in vitro in human hepatocytes [35]. However, lutein did not affect phase-I or phase-II liver enzyme activities in the rat [32]. Due to its close isomeric relationship to lutein, it is considered unlikely that zeaxanthin is a liver enzyme inducer.

In 2010, EFSA applied an additional safety factor of 2 in the ADI calculations for lutein [36] and in 2012 for zeaxanthin [37] to take account of the absence of chronic rodent studies.

### 3.6. ADME (Absorption, Distribution, Metabolism, and Excretion) Studies

Metabolism studies, or ADME studies, are a useful component of safety testing as the provided information on the metabolism and kinetics of a substance and, when human data is available, on human relevance.

#### 3.6.1. Balance Study in Rats

A distribution study with (^14^C)-zeaxanthin was performed in male rats, after a pretreatment feeding with zeaxanthin-poor or zeaxanthin-enriched diet (0.001% in feed) and subsequent single dose administration of (^14^C)-zeaxanthin in a liposomal preparation. One day after dosing, approximately two-thirds of the administered radioactivity was excreted in feces and urine and approximately 1/3 of the administered radioactivity was present in the body and GI-tract. The pattern of distribution in the tissues and excretion was similar for rats prefed with zeaxanthin-poor and those fed with zeaxanthin-enriched diet. After 1 week, less than 1% of the administered radioactivity was in the body and the digestive tract. The amount of radioactivity absorbed and excreted in the urine tended to be lower for animals fed with the zeaxanthin-poor diet. It was concluded that the radioactivity from (^14^C)-zeaxanthin is rapidly depleted from the body and the GI-tract of rats [38].

#### 3.6.2. Distribution Study in Rats

A study was performed to investigate zeaxanthin distribution in rats fed with a zeaxanthin 5% beadlet formulation-enriched diet. Male rats received a diet containing 10 mg or 100 mg zeaxanthin/kg feed (approximately 0.8 mg or 8 mg/kg bw/day) for five weeks. A dose-dependent accumulation of zeaxanthin was found in various tissues with the highest concentrations in the small intestine and spleen, followed by liver, fat, and adrenal glands. The thyroid gland and the eye levels were below the levels of detection. There was a marked decrease of zeaxanthin concentration during a subsequent 5-week reversibility period [39].

#### 3.6.3. Radioactivity in Expired Air, Mass Balance Study

In balance studies with a liposomal preparation of (^14^C)-zeaxanthin in male rats, about 1% of the administered dose, that is, about 4% to 7% of the absorbed dose, was measured in the expired air during the first 24 hours after administration. Contribution of respiration in the excretion of radioactivity was considerably higher in the case of zeaxanthin when compared to previous studies with astaxanthin and canthaxanthin. Absorption (biliary excretion not considered) varied from around 10% to around 20% [38, 40].

#### 3.6.4. Metabolite Studies

It is known that *β*-carotene is metabolised by central cleavage by the enzyme *β*, *β*-carotene-15, 15′-monooxygenase (CMO1). On the other hand, the nonprovitamin A xanthophylls, lutein, and zeaxanthin are metabolised preferentially by eccentric cleavage by carotene-9, 10-monooxygenase (CMO2), alternatively known as *β*-carotene oxygenase 2 (BCO2). *β*-Cryptoxanthin is also metabolised eccentrically through CMO2, which has been identified in humans, mice, and ferrets [41].

**Figure 2:**
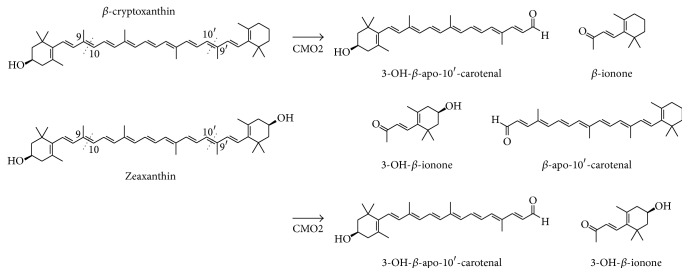
Xanthophyll carotenoids *β*-cryptoxanthin and zeaxanthin, and the metabolites from CMO2 cleavage, in ferrets (adapted from Mein et al., 2011) [42].

The metabolite pathways of lutein, zeaxanthin, and *β*-cryptoxanthin have been published in 2011 [42]. In this publication, the production of apocarotenoids from CMO2 metabolism in ferrets was shown for all of these three xanthophylls. All three are cleaved at the 9, 10 position as well as at 9′, 10′. This gives rise to four metabolites for both lutein and *β*-cryptoxanthin. Zeaxanthin however is symmetrical such that there are only two metabolites, 3-OH-*β*-apo-10′-carotenal and 3-OH-*β*-ionone. Both of these metabolites are derived from eccentric cleavage of lutein and *β*-cryptoxanthin (Figure 2). As zeaxanthin has the same ring structure at each end of the molecule, the same two metabolites are produced irrespective of whether cleavage occurs at the 9, 10 position or the 9′, 10′ position.

In addition to cleavage reaction products, there is evidence of a common metabolite from both lutein and zeaxanthin from noncleavage metabolism. In a human study with zeaxanthin, the metabolite all-E-3′-dehydro-lutein was formed; under normal dietary conditions, all-E-3′-dehydro-lutein is predominantly formed from other sources, most likely from lutein, rather than from dietary zeaxanthin [43].

Further, using chiral-phase HPLC, two diastereoisomers, (3R, 6′R)-3′-dehydro-lutein and (3R, 6′S)-3′-dehydro-lutein, were identified and shown to be common metabolites of lutein and zeaxanthin in rhesus monkeys [5].

### 3.7. Special Toxicological Studies

#### 3.7.1. One-Year Chronic Study in Monkeys

Safety studies are not normally undertaken in monkeys, at least not for nutritional substances. However, a known profile of human response that has been observed in the past in humans, with high intake of the carotenoid canthaxanthin, is accumulation in the eye and for the so-called “canthaxanthin retinopathy” [10]. Therefore, a chronic study was undertaken in monkeys with the purpose to assess the chronic safety of zeaxanthin and lutein in primates and to determine the potential for crystal formation in the retina. There are morphological differences in the structure of the eye between rodents and primates and further investigation in primates was considered important. There is no specific OECD guideline for the study design undertaken, which was designed taking into account general requirements for primate safety studies.

The chronic study performed in Cynomolgus monkeys was 52 weeks in duration and was an investigation for zeaxanthin and also for lutein (each with separate groups). 10% beadlet formulations of both substances were used. Oral doses of 0.2 and 20 mg/kg bw/day of zeaxanthin or lutein were given respectively by gavage to groups of 2 male and 2 female monkeys. For both 20 mg/kg bw/day groups, one additional male and female were sacrificed after 6 months of treatment. Normal toxicological endpoints were included as well as specific endpoints for the eyes.

All monkeys survived the treatment period. There were no clinical signs of toxicity and there was no effect of treatment on overall mean body weight gain or group mean food intake. At the high dose of zeaxanthin, orange-yellow coloration of the feces was observed during the treatment period and, at necropsy, yellow discoloration of adipose tissue was observed. These were considered as coloration changes from the presence of the test compound and were not considered an adverse effect. There were no changes in ECG or blood pressure data, considered to be related to zeaxanthin treatment. There were no treatment-related changes in urine, hematological, and serum clinical chemistry parameters. At necropsy, there were no abnormal gross findings or organ weight change. There were no treatment-related histopathological findings.

A comprehensive examination of the eyes of treated monkeys was performed which included ophthalmoscopy and biomicroscopy examinations, fundus photography, and electroretinography (ERG). Postmortem examinations of the retina of the right eye included macroscopic inspection, microscopic pathology under polarized and bright light, for peripheral retina and macula, confocal microscopy of macula, and histopathological examination of the peripheral retina. A determination of lutein and zeaxanthin in retina and lens of the left eye was performed by HPLC. These procedures and results are described in more detail.

*Ophthalmic Examinations*. Ophthalmic examinations were performed on the monkeys by two independent examiners. Indirect ophthalmoscopic examinations were performed using the Bonnoskop and direct ophthalmoscope and a contact lens biomicroscope. Overall, based on the ophthalmic examination findings, it was concluded that there were no adverse findings that were considered to be related to treatment and there was no evidence for crystalline deposits in the retina of treated monkeys [44, 45].

Additional evaluations were performed using the ophthalmic slit lamp biomicroscope in combination with wide-field corneal contact fundus lenses. The results of these examinations showed that there were no crystalline deposits or inclusions similar to those that have been seen in humans or in Cynomolgus monkeys ingesting high dosages of the carotenoid, canthaxanthin. There were some retinal findings often seen in the human- and nonhuman-primate retina; however, none of these were considered to be related to treatment [44, 45]. The polarising structures that were observed were found not only in the zeaxanthin- and lutein-treated monkeys, but also in the control monkeys. The implication of the special eye examinations included into the 52-week monkey study is considered to be that even for high intake zeaxanthin or lutein consumers; there is no indication that crystalline deposits could develop in the retina, as was seen in man and monkeys with high-dose canthaxanthin supplementation. 

*Electroretinography (ERG)*. ERG was performed in all animals once predose and during weeks 25 to 26, weeks 38 to 39, and weeks 51 to 52 of treatment. There were no treatment-related effects in electroretinograms, which is considered a sensitive procedure to detect early signs of generalized retinal degeneration [46]. 

*Eye Pathology*. Whole-mounts of retinas from the right eyes were used for microscopic investigations with light or confocal microscopy. Maculas were punched out with a 7 mm trephine before mounting them on slides and the peripheral remaining parts of the retinas were flat-mounted and investigated under the polarization microscope separately. Semiquantitative analysis of inclusions was performed by screening the flat-mounted retinas of the right eyes under polarized light using a Zeiss Axioplan. In addition, all maculas were investigated using a confocal microscopic system. Routine histopathology of paraffin sections from retinal periphery was performed [44, 45, 47]. The routine histopathological investigation of paraffin sections from retinal periphery did not show any differences between treated or control animals.

It was concluded that there were no treatment-related adverse changes in the eyes noted under the conditions of this study. Polarizing inclusions were observed in the macula of monkeys, which were not related to zeaxanthin nor lutein treatment. The incidence and grade of the inclusions in the maculas of the monkeys were not treatment or dose related. The inclusions clearly differed from crystals observed after long-term treatment at high doses of canthaxanthin. In the case of canthaxanthin, crystals were strongly dose-dependent, occurred predominantly in the peripheral retina, and exhibited crystalloid morphology and larger size [12]. In contrast, inclusions in the current study were restricted to the fovea, were very small, and showed no typical crystalline morphology. The nature of the observed polarizing structures remains unknown. Since they were also observed in control animals with a naturally yellow macula, a physiological function may be hypothesized [48]. 

*Zeaxanthin and Lutein Determinations in Retina and Lens by HPLC*. Determination of lutein and zeaxanthin in retina and lens was made using HPLC. Treatment with lutein resulted in a dose-related increase of lutein in central retina, peripheral retina, and lens. In addition, after treatment with lutein at both dose levels, elevated amounts of zeaxanthin were observed in the central retina. This finding may be due to the residual zeaxanthin content in the lutein test article. Zeaxanthin levels in peripheral retina and lens were similar to those observed in the placebo group [49].

Treatment with zeaxanthin resulted in a dose-related increase of zeaxanthin in the peripheral retina. In central retina and lens, zeaxanthin content was markedly increased in animals of the high-dose group. Levels in the low dose group were comparable to those determined in the placebo group. In animals treated with zeaxanthin, lutein content was in the same order of magnitude as in the placebo group [49].

Variability of individual animal lutein and zeaxanthin content was considerable in all tissues investigated for both sexes and at all dose levels including the placebo group. Considering the variability, there was no significant difference between sexes. In addition, no relevant difference was observed in animals sacrificed in week 26 and animals sacrificed at the end of the treatment period. This suggests that steady state conditions were reached before week 26 in all eye segments investigated.

Overall, there were no clinical and no morphological evidence for treatment-related adverse changes in the eyes of Cynomolgus monkeys during or after 52 weeks of treatment with zeaxanthin or lutein, both as a 10% beadlet formulation. Specifically, there was no evidence for crystal formation in the eyes of treated monkeys. The NOAEL for lutein and for zeaxanthin was the highest dosage, 20 mg/kg bw/day.

#### 3.7.2. Other Monkey Studies

There is a published study with female rhesus macaques (5/group) exposed to 10 mg/kg bw/day of lutein supplements providing 9.34 mg lutein and 0.66 mg zeaxanthin, 10 mg/kg bw/day of zeaxanthin supplements, or supplements of a combination of lutein and zeaxanthin (each at 0.5 mg/kg bw/day) for 12 months [50]. After 12 months, one control animal, two lutein-treated animals, two zeaxanthin-treated animals, and all lutein and zeaxanthin combined-treated animals were killed. The other animals were kept under observation for six additional months without receiving further supplementation and were then killed. Plasma and ocular carotenoid analyses, fundus photography, and retina histopathology were performed on the animals.

Supplementation of female rhesus macaques with 9.34 mg lutein/kg bw/day or 10 mg zeaxanthin/kg bw/day for 12 months resulted in 3.2-fold and 3.7-fold increases in the mean concentrations of lutein and 4.0-fold and 4.3-fold increases in the mean concentrations of zeaxanthin, in plasma and retina, respectively. Supplementation of monkeys with lutein or zeaxanthin for one year at a dose of approximately 10 mg/kg bw/day did not cause ocular toxicity and had no effect on biomarkers associated with nephrotoxicity.

### 3.8. Inhalation Study in Ferrets

No carcinogenic hazard is expected from direct intake of zeaxanthin or lutein. There has been a question as to whether these xanthophylls might exacerbate the risk of lung tumors in heavy smokers as was indicated to occur in two human intervention studies with high dosages of *β*-carotene [51, 52]. It has been established that this exacerbating influence of *β*-carotene could be mimicked in the ferret [53], a species selected on the basis of metabolic considerations and certain similarities to man. Ferrets show a weak central (CMO1) cleavage of *β*-carotene in a similar way to humans. In contrast, rats show a much stronger CMO1 activity and a greater propensity to centrally split beta-carotene, which raised doubts about the relevance of the rat as a suitable human model.

This concern of a possible adverse influence in combination with smoking can potentially be addressed for zeaxanthin using a published study in ferrets treated with *β*-cryptoxanthin and exposed to cigarette smoke [54]. Zeaxanthin itself has not been tested in the ferret model. Structurally, zeaxanthin is closely related to *β*-cryptoxanthin. As described previously, the metabolites of zeaxanthin central cleavage, 3-OH-*β*-apo-10′-carotenal and 3-OH-*β*-ionone (Figure 2), are also metabolites of *β*-cryptoxanthin central cleavage. From this overlap of CMO2 metabolites and as CMO1 in the lung of man and the ferret is not the predominant cleavage enzyme, data from the *β*-cryptoxanthin study can contribute to zeaxanthin evaluation on a “read-across” basis.

In this *β*-cryptoxanthin study, both the low and high dose lowered the incidence of cigarette smoke-induced lung squamous metaplasia. The reduction was significant for the high dose (*p* = 0.015, 1/6 ferrets affected) and was marginally significant for the low dose (*p* = 0.06, 2/6 ferrets affected), compared to the control (6/6 ferrets affected). Further, the expression of proinflammatory markers TNF*α* (expression of which was tremendously increased in smoke exposed ferret lungs) and of NF-*κ*B was lowered by *β*-cryptoxanthin administration, with stronger beneficial effects for high-dose *β*-cryptoxanthin than for the low-dose *β*-cryptoxanthin.

However, the usefulness of this read-across approach was limited by the dose selection in the *β*-cryptoxanthin ferret study. The dosages of *β*-cryptoxanthin used (7.5 *μ*g/kg and 37.5 *μ*g/kg bw/day) were based on equivalence to an average American intake of 104 *μ*g/day (approximately 1.5 *μ*g/kg bw/day for a 70 kg person) increased by a factor of 5 and 25, and not by a factor of at least 100, as is usual in toxicological safety testing. Also from a read-across perspective, only half of the CMO2 metabolites formed from *β*-cryptoxanthin would be theoretically common to those from zeaxanthin. So ignoring any possible kinetic differences, 37.5 *μ*g/kg bw/day possibly only corresponds to 18.75 *μ*g/kg bw/day in terms of zeaxanthin dosage, or 1.3 mg/day for a 70 kg adult. The relative “internal” human dose could be even lower if systemic carotenoid absorption in the ferret is lower than in man, as indicated by the authors [54].

So from this study with *β*-cryptoxanthin in ferrets, it is considered that zeaxanthin supplementation at low intakes is unlikely to exacerbate the occurrence of lung cancer and might even have a protective effect against the occurrence of squamous metaplasia. However, due to the low dosages of *β*-cryptoxanthin used, the extent to which the dosage-related influences might extend to higher intakes of *β*-cryptoxanthin or intakes of zeaxanthin above 1.3 mg/day is unclear.

## 4. Summary and Discussion 

A series of well-conducted safety studies are available and provide a good basis for a safety assessment of zeaxanthin. Acute studies in rats and mice show a low order of acute toxicity with LD_50_ values greater than 4000 and 8000 mg/kg, respectively. Subchronic safety studies demonstrated that repeated intakes of high oral doses up to 1000 mg/kg bw/day in rat and mouse and 400 mg/kg bw/day in the dog are well tolerated systemically. The macroscopic observation of yellow discoloration of the adipose tissue, which can be attributed to the presence of the zeaxanthin, indicates that there was systemic exposure in these studies and this has been analytically confirmed by analysis of plasma and liver samples in the two-generation rat study. Despite the systemic exposure and high dosages, no target organ toxicity was identified in the subchronic studies during the in-life phase or by pathological/histopathological evaluation.

In developmental toxicity studies, there was no evidence of maternal toxicity, fetal toxicity, or teratogenicity in treated rats or rabbits at doses up to 1000 mg/kg/day and 400 mg/kg/day, respectively.

For zeaxanthin, there is no chronic study in rodents, as is also the case for lutein, but there is a two-generation study. This study design involves exposure to test compounds beginning before mating, continuing during mating and throughout gestation and lactation, until weaning, and covers all reproductive life phases over two generations. The new version of the study design finalized in 2001 introduced a range of additional end-points focused on detection of fine disturbances of reproductive function and fertility. Such studies can sometimes give a lower NOAEL than respective subchronic toxicity studies, in a similar way that the NOAELs from chronic studies in general are lower than in corresponding subchronic studies. Indeed, on the basis of the dosages for which data was available, the NOAEL from the rat two-generation study with zeaxanthin (150 mg/kg bw/day) was a factor of 6.7 down from the NOAEL in the subchronic rat study.

Potentially, the high systemic exposure observed in young animals during the lactation phase of the two-generation study, from a combination of intake from maternal milk and direct feeding, may have contributed to an effect of treatment occurring at a lower nominal dosage than in the other safety studies. Irrespective of the reason, the lowest dosage from repeat dose toxicity studies was the intermediate dosage of 150 mg zeaxanthin/kg bw/day.

ADME studies in the rat showed that zeaxanthin was rapidly but incompletely absorbed after oral administration following a single dose of ^14^C-zeaxanthin and there is a wide bodily distribution with clear deposition in fatty tissues reflecting the lipophilic nature of zeaxanthin. In addition to specific ADME studies, important information on the potential to bioaccumulate can be obtained from samples taken during the course of the toxicology studies. As referred to in the two-generation study, there is evidence of accumulation in the liver in comparison to concentrations in the plasma.

Metabolite studies have shown there is eccentric CMO2 cleavage of zeaxanthin and other xanthophylls but for zeaxanthin, being symmetric, only two rather than four metabolites are expected. Both CMO2 cleavage metabolites of zeaxanthin occur as cleavage metabolites of lutein and *β*-cryptoxanthin.

Genotoxicity studies are important studies to indicate if there is interaction with DNA. When unformulated pure crystalline zeaxanthin is exposed to air and light, there may be a potential for mutagenic breakdown products to occur. However, DSM formulated zeaxanthin contains antioxidants that prevent the degradation of zeaxanthin. No mutagenicity was observed in the Ames test with zeaxanthin, or crystalline zeaxanthin retained beyond the shelf life, or in cultures of V79 at the HGPRT locus. No evidence of Unscheduled DNA Synthesis was detected in rat hepatocytes up to the highest dose tested. There was no evidence of clastogenic potential with or without metabolic activation from tests with peripheral blood lymphocytes at doses. In the* in vivo* mouse micronucleus test, there was no evidence for mutagenicity or clastogenicity. It is concluded that there is no evidence for mutagenicity or clastogenicity with formulated zeaxanthin under appropriate conditions of use.

Based on the wide range of genotoxicity studies with no indication of DNA damage and the absence of any indication of preneoplastic organ changes in repeat dose toxicity studies, and the absence of clear liver enzyme induction effect for lutein, no carcinogenic hazard is expected from direct intake of zeaxanthin.

The question as to whether these xanthophylls might exacerbate the incidence of lung tumors in heavy smokers, as was demonstrated to occur for high dosages of *β*-carotene in two human intervention studies [51, 52], has only been partly addressed by using published studies in ferrets for this related xanthophyll. The ferret model with exposure to cigarette smoke has been positively validated for *β*-carotene [53] and the metabolite overlap has enabled theoretical use of a study with *β*-cryptoxanthin to support the safety of zeaxanthin, on a read-across basis. Although the study showed a protective effect of *β*-cryptoxanthin against pulmonary squamous metaplasia, the potential applicability of this data for zeaxanthin intake was considered to be limited, due to the low *β*-cryptoxanthin dosages that were used in the study.

Besides this study in ferrets, a pooled analysis of seven cohort studies demonstrated that the association between intake of xanthophylls (lutein or lutein plus zeaxanthin) and the risk of lung cancer was negative in smokers and nonsmoking subjects [55].

In general, the structural similarity of the xanthophyll compounds might be considered sufficient to enable the principles of read-across, where there are safety data gaps, as has been done with the *β*-cryptoxanthin data in ferrets. However, it appears to be the case that, in the human and primate eye, there is a notable specificity in biological differentiation between the xanthophyll isomers [56]. With this being the case, there is the need for caution in carrying across information from one of these related substances to another, at least in respect to the primate eye.

The animal safety data for lutein and meso-zeaxanthin is notably less than what is available for zeaxanthin. EFSA has reviewed the available data for lutein [36]. For meso-zeaxanthin, there is published safety information [57]. In genotoxicity studies reported by Xu et al., there was no evidence of genotoxicity, which is consistent with the data for zeaxanthin. The NOAEL from their 13-week rat toxicity study was 300 mg/kg bw/day with clear adverse effects in the liver being reported at the higher dosages of 600 and 1200 mg/kg bw/day. This is in contrast to the subchronic safety data for synthetic zeaxanthin, where higher NOAELs were obtained with no indication of liver toxicity. No published regulatory 13-week rat study with *β*-cryptoxanthin could be located although there is ADME data in the rat following chronic oral intake [58].

There is a publication reporting toxicology studies for a lutein and zeaxanthin concentrate from marigold flowers (*Tagetes erecta L.*), with a minimum 80% carotenoid content [59]. In the subchronic study, Wistar rats were administered the concentrate at dose levels of 0, 4, 40, and 400 mg/kg bw/day (gavage) for 13 weeks with no toxicologically significant treatment-related changes. The dosage in terms of zeaxanthin, at the high dose, can be calculated to be 21.6 mg/kg bw/day (taking 7.5% of the carotenoid content to be zeaxanthin).

In a recent publication of safety studies of the zeaxanthin concentrate OmniXan, RR-zeaxanthin 65% enriched product obtained from paprika [60], there was no indication of genotoxicity. In the 13-week rat toxicity study, the highest dosage in terms of concentrate was 400 mg/kg bw/day and this was considered the NOAEL.

A known profile of human response that has been observed in the past in humans, with a high intake of the carotenoid canthaxanthin, is accumulation in the eye and for so-called “canthaxanthin retinopathy.” The accumulation in the eyes, however, was not found to be functionally harmful and gradually reversible following discontinuation of consumption. Nevertheless, this accumulation is regarded as undesirable and has been evaluated as an adverse effect by EFSA [61].

A chronic study with synthetic zeaxanthin in Cynomolgus monkeys, an animal model used to investigate the induction and dose dependency of canthaxanthin crystal formation in the retina, has been undertaken involving a comprehensive battery of ocular testing as well as usual toxicological endpoints. Overall, there were no clinical and no morphological evidence for treatment-related adverse changes in the eyes and specifically no evidence for crystal formation in the eyes of treated monkeys.

In the safety evaluation of dietary substances, including nutritional substances being consumed at higher intakes than traditionally occurs, human data needs to be kept in mind as it becomes available. A number of human intervention studies have been undertaken or are in progress with respect to investigating the protective function for zeaxanthin and lutein in the eye. These studies indicate good systemic tolerance of zeaxanthin. At the upper end of the dosage range were a study with a dose of up to 20 mg/day for up to 6 months [62] and a study with 8 mg/day for a year, both without evidence of adverse effects. A further study has been more recently reported in which 24 subjects were supplemented with 20 mg/day of zeaxanthin over 4 months, without any adverse effects [63, 64].

### 4.1. ADI (Acceptable Daily Intake)

In the 13-week subchronic toxicity studies, the NOAEL in all cases was the highest dosage investigated, namely, 1000 mg/kg bw/day in the mouse and rat and at least 422 mg/kg bw/day in the dog. A traditional approach of a 100-fold safety factor in conjunction with the lowest relevant NOAEL from the safety studies would be used to derive the ADI.

For zeaxanthin, the lowest NOAEL from a standard regulatory study was 150 mg zeaxanthin/kg bw/day in the two-generation study in rats. This NOAEL is at least a factor of 6.7 lower than the NOAEL in the 13-week rat study (>1000 mg/kg bw/day). In their evaluation of the safety of synthetic zeaxanthin as a Novel Food, the EFSA Panel on Dietetic Products, Nutrition, and Allergies (NDA) [37] used the 150 mg/kg bw/day NOAEL with a 200-fold safety factor to define an ADI of 0.75 mg/kg bw/day, or 53 mg/day for a 70 kg adult (70 kg is the new default human weight used by EFSA). Use of the lowest NOAEL for ADI calculations, as was done by the NDA Scientific Panel, is the traditional precautionary approach used in safety evaluation. The NDA Scientific Panel stated that a daily intake of 53 mg for a person with a body weight of 70 kg does not raise safety concerns and that the use level of 2 mg/day requested by the applicant was confirmed as safe.

In the case of lutein, the EFSA Panel on Food Additives and Nutrient Sources added to Food (ANS) in their reevaluation [36] introduced an additional safety factor of 2 (making a total 200-fold safety factor), due to the absence of chronic studies or a multigeneration reproductive toxicity study. Additional factors taken into account were that the other data (reproductive studies and genotoxicity data) did not indicate a cause for concern and that lutein is a normal constituent of the diet. The highest dose tested for lutein in a comprehensive 13-week rat toxicity study was 200 mg/kg bw/day and this was the NOAEL. The EFSA ANS Panel applied a 200-fold factor to this NOAEL giving an ADI of 1 mg/kg bw/day or 60 mg lutein/day for an adult.

As a passing comment, application of the 200-fold factor to the rat zeaxanthin subchronic data would give a 5-fold higher ADI than for lutein, due to the higher dosages used and the higher NOAELs that were established for zeaxanthin.

In the United States, synthetic zeaxanthin is marketed since 2002 under the Generally Regarded as Safe legislation, based on DSM safety studies available at the time, with use level in foods and beverages of 0.25 mg/serving.

The use level of zeaxanthin of 2 mg/day proposed by the applicant was ratified by the European Union (EU) Commission in 2013 [65]. However, this upper use level is much lower than the safe level (53 mg/day) defined for zeaxanthin by the NDA Scientific Panel. Potentially, therefore, the currently approved level for synthetic zeaxanthin in Europe could be set as a higher level. Probably, this could be closer to the ADI calculated by the NDA Panel. This ADI (53 mg/day) is similar to the ADI of 60 mg/day currently defined by EFSA for lutein [36].

## 5. Conclusion

Zeaxanthin was negative for mutagenic and clastogenic activity in a comprehensive battery of* in vitro* and* in vivo* tests for genotoxicity. Based on these studies, it is concluded that there is no evidence for mutagenicity or clastogenicity with formulated zeaxanthin under appropriate conditions of use.

In repeat dose toxicity studies in the rat, mouse, and dog, synthetic zeaxanthin was well tolerated at high dosages with no indication of target organ toxicity or preneoplastic organ changes. Taken together, these data indicate that no carcinogenic hazard is expected from direct intake of zeaxanthin. A study in primate did not indicate any evidence of ocular toxicity or excessive accumulation. A published study in ferret provides limited support for the absence of any stimulating effect of zeaxanthin consumption on the incidence of lung cancer in heavy smokers.

The regulatory study that gave rise to the lowest overall NOAEL of 150 mg zeaxanthin/kg bw/day was a comprehensive two-generation study in the rat. In their evaluation of the safety of synthetic zeaxanthin as a Novel Food, the EFSA NDA Scientific Panel [37] applied a 200-fold safety factor to this NOAEL to define an ADI of 0.75 mg/kg bw/day, or 53 mg/day for a 70 kg adult. The EU in 2013 [65] formally approved upper use levels of 2 mg/day (equivalent to 0.03 mg/kg bw/day) as this was the use level proposed by the applicant.

Information from human intervention studies also supports that an intake higher than 2 mg/day is safe, and an intake level of 20 mg/day for up to 6 months was without adverse effect.

## Figures and Tables

**Table 1:** Average intake of lutein and zeaxanthin by age group (Mohamedshah et al*.,* 1999) [3].

Age group	Lutein (*μ*g/day)	Zeaxanthin (*μ*g/day)	Lutein : Zeaxanthin ratio
20–29	745	178	4.2 : 1
30–39	896	174	5.1 : 1
40–49	920	187	4.9 : 1
50–59	1053	182	5.8 : 1
60–69	1056	170	6.2 : 1
70+	990	170	5.8 : 1

**Table 2:** List of genotoxicity, repeat dose, and reproductive safety studies conducted with DSM-manufactured synthetic zeaxanthin based on international regulatory study designs.

Safety studies	Formulation nominal %	Concentration or dosage	Result
		Concentration	

Genotoxicity *in vitro*			
Ames, *S. typhimurium* mutation assay	Crystalline	0, 2.4–1500 *μ*g/plate	Negative
Gene mutation in V79 cells	Crystalline	0, 1–16 *μ*g/mL	Negative
Unscheduled DNA Synthesis (UDS) in rat hepatocytes	Crystalline	0.1–16 *μ*g/mL	Negative
Human lymphocytes	Crystalline	0, 60, and 120 *μ*g/mL	Negative

		Dose (mg/kg bw/day)	

Genotoxicity assays *in vivo*			
Mouse micronucleus	10% beadlet	0, 44.5, 89, and 178	Negative
Subchronic and chronic			
13-week oral (admix) in mice	10% beadlet	0, 0, 250, 500, and 1000	NOAEL, high dose
13-week oral (admix) in rats	10% beadlet	0, 0, 250, 500, and 1000	NOAEL, high dose
13-week oral (feed cubes) in dogs	10% beadlet	0, 123, 204, and 422 males : 0, 104, 238, and 442 females	NOAEL, high dose
1-year oral (gavage) in monkeys zeaxanthin or lutein	10% beadlet	0, 0.2, and 20 for zeaxanthin or lutein	NOAEL, high dose
Reproductive studies			
Teratology oral (admix) in rats	10% beadlet	0, 250, 500, and 1000	NOAEL, high dose
Teratology oral (gavage) in rabbits	Crystalline in oil	0, 100, 200, and 400	NOAEL, high dose
Two-generation (admix) in rats	10% beadlet	0, 0, 50, 150, and 500	NOAEL, inter. dose

**Table 3:** Effects on reproduction data in the zeaxanthin two-generation study in rats [31].

Nominal dosage (mg/kg bw/day)	0	0 (placebo)	50	150	500
F_1_ generation					
Adults, mating index %	100.0	100.0	96.0	96.0	79.3 ^*∗*^ *F*
Mean number of pups:					
born	10.2	10.9	10.7	10.7	9.7
alive day 4 *postpartum* (before culling)	10.0	10.9	10.5	10.5	9.7
P generation					
% pup weight gain					
Days 4–7 *postpartum*	62.3	62.0	64.2	61.6	59.3
Days 1–21 *postpartum*	774.7	753.3	780.9	755.0	725.3 ^*∗∗*^ *J*
F_1_ generation					
% pup weight gain					
Days 4–7 *postpartum*	64.4	62.8	60.5	61.3	58.1 ^*∗*^ *J*
Days 1–21 *postpartum*	738.9	745.1	713.5	724.9	695.0

*F* = Cochran-Armitage and Fisher's exact test.

*J* = dose response test, Kruskal-Wallis, Terpstra-Jonckheere, and Wilcoxon.

^*∗*^ *p* < 0.05; ^*∗∗*^ *p* < 0.01.

**Table 4:** Plasma and liver concentration of zeaxanthin in adults and weanlings in the two-generation study in rats [31].

Zeaxanthin concentration (*μ*/L or *μ*/kg) at nominal dosage (mg/kg bw/day)					
Dosage	0	0	50	150	500
*P generation*					
*Plasma*, *μ/L*					
Adults					
Male	—	—	24	47	111
Female	—	—	19	29	71
Weanlings	—	—	61	127	353
*Liver*, *μ/kg*					
Adults					
Male	14	—	599	1121	2581
Female	4	5	1147	1992	3159
Weanlings	—	—	4015	10892	23836

*F*_*1*_ * generation*					
*Plasma*, *μ/L*					
Adults					
Male	—	—	22	42	109
Female	—	—	20	42	87
Weanlings	—	—	52	85	213
*Liver*, *μ/kg*					
Adults					
Male	—	—	114	645	1689
Female	11	—	1077	1382	3785
Weanlings	—	11	4313	7904	21611
